# Risk Factors for Serious Bacterial Infections Among Young Infants With Hypothermia: Protocol for a Multicenter, Retrospective Case-Control Study

**DOI:** 10.2196/66722

**Published:** 2025-06-19

**Authors:** Sriram Ramgopal, Paul Aronson, Douglas Lorenz, Alexander Joseph Rogers, Andrea Tania Cruz

**Affiliations:** 1 Division of Emergency Medicine Ann & Robert H. Lurie Children's Hospital of Chicago Chicago, IL United States; 2 Department of Pediatrics, Section of Pediatric Emergency Medicine Yale School of Medicine New Haven, CT United States; 3 Department of Emergency Medicine, Section of Pediatric Emergency Medicine Yale School of Medicine New Haven, CT United States; 4 University of Louisville Louisville, KY United States; 5 Department of Emergency Medicine University of Michigan Ann Arbor, MI United States; 6 Department of Pediatrics University of Michigan Ann Arbor, MI United States; 7 Division of Emergency Medicine, Department of Pediatrics Baylor College of Medicine Houston, TX United States; 8 Division of Pediatrics, Department of Pediatrics Baylor College of Medicine Houston, TX United States

**Keywords:** hypothermia, serious bacterial infection, urinary tract infection, UTI, bacteremia, bacterial meningitis, herpes simplex virus, noninfectious pathologies, environmental factors, pediatrics, infants, neonatal, infectious diseases, bacterial infection, herpes

## Abstract

**Background:**

Hypothermia in young infants presenting to the emergency department (ED) may indicate a serious bacterial infection (SBI) such as a urinary tract infection, bacteremia, or bacterial meningitis. Improved understanding of the epidemiology of SBI in infants with hypothermia and the development of prediction models can help avoid unnecessary invasive procedures and antimicrobial exposure.

**Objective:**

The aim of the study is to (1) describe the epidemiology of SBI and herpes simplex virus (HSV) among infants with hypothermia, (2) assess the role of biomarkers in predicting SBI, and (3) derive and internally validate a multivariable predictive model for SBI among infants with hypothermia.

**Methods:**

The study is being conducted through the Pediatric Emergency Medicine Collaborative Research Committee as a retrospective nested case-control study. We will include infants with hypothermia (rectal temperature<36.5 °C) presenting to 1 of 28 pediatric EDs in the United States between January 1, 2013, and December 31, 2022. Exclusion criteria will include (1) fever in the ED or prior to ED arrival, (2) transfer from another health care facility, (3) technology dependence, (4) trauma, (5) skin and soft tissue infections, and (6) presentation in cardiac arrest. The primary outcomes will be culture-confirmed SBI (objectives 1-3) and HSV-positivity (objective 1). The analytic approach for each objective will be (1) to use descriptive statistics to summarize the epidemiology of SBI and HSV and clinical outcomes, (2) to compare biomarker parameters of infants with and without SBI and determine optimal cutoff values using a receiver operator characteristic curve, and (3) to develop a multivariable predictive model using penalized mixed effects logistic regression within a 1:3 case-control sample among infants in the cohort who had a blood culture obtained.

**Results:**

Data collection for this study is ongoing, with a collection of data from 21 hospitals at the time of protocol submission. Of 16 sites evaluated in preliminary analysis (n=45,673), the median age is 25 (IQR 6-52) days, and 24,182 (52.9%) are male. In total, 340 (0.7%) infants have an SBI, of whom 103 had bacteremia or meningitis. Mortality occurred in 7 (2.1%; 95% CI 0.9%-4.3%).

**Conclusions:**

We will use a consensus-based outcome measure for SBI with an established data acquisition pathway. We will use a multicenter sample from US children’s hospitals, using a consensus-based outcome measure for SBI and a case-control approach to evaluate outcomes to improve the management of young infants with hypothermia in the ED.

**International Registered Report Identifier (IRRID):**

DERR1-10.2196/66722

## Introduction

Globally, neonatal sepsis is estimated to occur in nearly 3000 cases per 100,000 live births, with an estimated mortality rate of 18% [1]. While abnormalities of temperature (including fever or hypothermia) are understood to be a risk factor for sepsis [2,3], the specific association of hypothermia with high-risk infections is poorly characterized. Hypothermia among young infants presenting to the emergency department (ED) may be a sign of a serious bacterial infection (SBI), which includes urinary tract infection (UTI), bacteremia, or bacterial meningitis [4,5]. Prior study suggests that hypothermia may also occur in disseminated herpes simplex virus (HSV) infection [6-8]. However, infants more frequently present with hypothermia due to noninfectious pathologies or secondary to environmental factors [9,10]. The etiology of hypothermia in the setting of sepsis is likely multifactorial, with some preclinical studies suggesting an advantageous role for hypothermia as compared to fever in rats with severe endotoxemia [11]. It remains unclear what percentage of infants with hypothermia are ill-appearing or have other relevant findings on examination. Prior work has provided limited data with respect to SBI among young infants with hypothermia. These studies, performed from single centers or from administrative datasets, have identified that SBIs occur in 1%-8% of young infants with hypothermia [5,6,9,12-15].

In contrast to young infants with fever [16,17], infants presenting to the ED with hypothermia lack prospectively derived evidence to guide care. In the absence of robust evidence-based consensus and guidelines to assist clinicians, substantial variability exists in the care provided for these infants, as demonstrated in one large survey of 1231 ED clinicians [18]. This is further challenged by the lack of a consistent definition for hypothermia, which varies from 36.0 °C to <36.5 °C [5,6,15]. Many infants with hypothermia are managed similarly to young infants with fever [18], but this approach has not been fully studied. There is wide variability in the performance of cerebrospinal fluid (CSF) testing (14%-70%), obtaining blood for inflammatory markers (8%-88%) and empirical antibiotic use (56%-92%) [19]. These data also suggested an increasing reliance on the use of inflammatory markers to guide decision-making, including C-reactive protein (CRP) and procalcitonin [19]. Nevertheless, prior work has demonstrated associations of clinical factors with the presence of bacterial infections among infants with hypothermia, including age [4,6], temperature [4,14], persistence of hypothermia [5], laboratory measures [4-6], and examination factors [9,12]. A well-performing prediction model to predict SBI is currently lacking.

Improved data are required to evaluate the prevalence of SBI among young infants with hypothermia and to construct a predictive model for this outcome. This will help identify infants at very low risk for SBI in whom invasive procedures and unnecessary antimicrobial exposure can be avoided. This study therefore has the following three objectives: (1) to describe the epidemiology and prevalence of SBI and HSV among young infants (≤90 days) presenting to the ED with hypothermia, (2) to evaluate the role of biomarkers in the prediction of SBI among young infants, and to derive and internally validate a prediction rule for SBI among young infants presenting to the ED with hypothermia.

## Methods

### Overview

**Figure 1:**
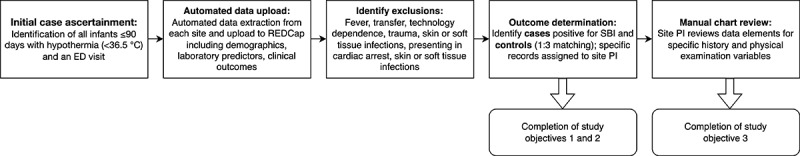
Overall flowchart of study protocol. ED: emergency department; PI: principal investigator; REDCap: Research Electronic Data Capture; SBI: serious bacterial infection.

We will perform a multicenter study of infants with hypothermia in the ED using the Pediatric Emergency Medicine Collaborative Research Committee (PEM CRC) to characterize infants with SBI and HSV and to compare infants with hypothermia with and without SBI. The PEM CRC offers a framework for multicenter investigations, supporting and facilitating the development of valuable and rigorous collaborative research in pediatric emergency medicine. PEM CRC plays a crucial role in developing high-quality protocols and identifying interested coinvestigators across its member sites. The PEM CRC has established a centralized data center at Texas Children’s Hospital, Baylor College of Medicine (BCM). This center offers data collection support services, including front-end logic checks for electronic data sheets, high-speed automated data sheet scanning, and database creation, encryption, and processing, and has conducted several important studies focused on topics relevant to the care of uncommon, but clinically important, conditions that occur in children presenting to the acute care setting [20-23]. We will include infants ≤90 days with a blood culture performed and who presented to a contributing hospital with a rectal temperature of <36.5 °C in the ED over a 10-year period. We will use a case-control approach. BCM will serve as the data coordinating center. A graphical summary of the study protocol is provided in Figure 1.

### Approaches

#### Objective 1 Approach

We will construct a large, retrospective, multicenter dataset assembled from at least 25 participating hospitals to identify the frequency of presentation of hypothermia across participating hospitals, the incidence of SBI and HSV, types of SBI, the underlying bacteriology of SBI, and clinical outcomes of infants with SBI, including in-hospital mortality. We will describe secondary outcomes of infants to describe diagnoses and evaluate clinical outcomes of hospitalization, length of stay, use of the intensive care unit, diagnoses, and unscheduled return visits to the ED following ED discharge.

#### Objective 2 Approach

We will analyze infants with hypothermia who do and who do not have SBI to identify the predictive value of frequently performed tests and calculate ideal cutoffs for these tests to maximize their diagnostic value. As an exploratory component of this aim, we will additionally investigate the role of newer tests of inflammation for SBI in this population, including CRP and procalcitonin.

#### Objective 3 Approach

We will construct a prediction model using a multicenter dataset. We will use penalized multivariable logistic regression to develop a full model and bootstrapped backward selection models to develop a parsimonious reduced model. A prediction rule for infants with hypothermia will allow for the identification of infants at low risk of SBI. The identification of historical, clinical, and laboratory risk factors would facilitate real-time risk stratification and identification of a low-risk group. The performance and reporting of this prediction model will adhere to the Transparent Reporting of a Multivariable Prediction Model for Individual Prognosis or Diagnosis guideline [24].

### Inclusion Criteria

Infants ≤90 days of age presenting to the ED of a participating pediatric ED with hypothermia, defined as a minimum rectal temperature of <36.5 °C in the ED over a 10-year period (January 1, 2013, to December 31, 2022). The <36.5 °C criterion is determined based on preliminary work performed by the investigators [14] and uses the World Health Organization criteria to define hypothermia in neonates [25]. We will allow participating sites that are unable to provide data from 2011 to contribute data due to varying adoption of EHR systems across these hospital systems [23].

### Exclusion Criteria

The exclusion criteria are as follows:

1. Fever in the ED or prior to ED arrival. Fever is recognized as a risk factor for SBI among young infants presenting to the ED [4]. Infants with fever should be managed according to national guidelines recommending routine testing for infants with fever on ED arrival. We will use a threshold for fever (rectal temperature ≥38.0 °C) used by the American Academy of Pediatrics in a clinical practice guideline published for the evaluation of this condition [26].
2. Transfer from another health care facility. It is difficult to accurately assess or obtain assessment or laboratory data present at the time of initial outside health care facility evaluation; as such, we exclude infants who were transferred to a study site from a different ED or hospital.
3. Technology dependence. We will identify infants with technology dependence (eg, ventriculoperitoneal shunts) using the ICD-10 (International Statistical Classification of Diseases, Tenth Revision) procedure and diagnosis codes documented for the patient during any prior encounter within the hospital electronic health record (EHR) system. These will be identified using the technology dependence flag of the pediatric chronic complex condition algorithm [27].
4. Trauma. Hypothermia can occur in the setting of traumatic injury, including child abuse [28]. Children with an encounter-level diagnosis consistent with trauma will be excluded using
  ICD-10
  codes from a validated injury diagnosis framework [29].
5. Skin and soft tissue infections. Children with skin and soft tissue infections frequently demonstrate a clinically apparent source of infection at the time of presentation. While these are known to occur among young infants with fever [30], the incidence of skin and soft tissue infections among infants with hypothermia is unknown. These infections will be identified using the encounter-level ICD-10 codes L030, L031, L032, L033, L038, and L039 [31].
6. Presentation in cardiac arrest. Children may be hypothermic because they are present in a perimortem state. We will specifically exclude children who present to the ED in cardiac arrest or postmortem state. These will be identified by finding all infants who received cardiopulmonary resuscitation during their hospitalization, had a diagnosis code consistent with cardiac arrest, or had in-ED or in-hospital mortality. This process is intended to be sensitive to identify all potential encounters with cardiac arrest. We will ask each site principal investigator (PI) to manually review these cases to identify those presented in cardiac arrest or for whom resuscitative efforts were not attempted.

As our inclusion period includes the period of time prior to the adoption of *ICD-10* in the United States, we will crosswalk the *ICD-9* (*International Classification of Diseases, Ninth Revision*) diagnosis codes using generalized equivalence mappings, a resource developed by the US Centers for Medicare & Medicaid Services that provides forward and backward mappings between the diagnosis coding systems [32].

### Case Ascertainment and Initial Data Upload

The EHR will be queried each participating ED to identify eligible age (≤90 days) encounters from 2013 to 2022 with at least 1 documented episode of hypothermia during the ED encounter. Due to the large volume of records anticipated to be identified at each site (varying between 1500 and 3000 encounters on initial efforts from 7 hospitals), data collection will proceed through 2 phases to maximize the efficiency of manual chart review and avoid the collection of unnecessary data. First, we will use a standardized data collection of all variables that can be ascertained from the EHR through computerized queries. This will include (1) demographic details such as age, race, ethnicity, and sex, and administrative data (payor status, year, and season of visitation); (2) medical history, ED chief complaints, multiple temperature measurements throughout the visit, and heart rate and blood pressure data; (3) laboratory test results, including complete blood counts (CBCs), metabolic panels, and tests for viral and bacterial pathogens (including the results of urine, blood, and CSF culture, results from urinalysis and urine dipstick testing, and results from HSV testing); (4) results from chest radiography, if performed; (5) medication administrations (antipyretics, antibiotics, and antiviral medications); (6) ED outcomes and details of hospitalization such as neonatal or pediatric intensive care unit admissions; (7) follow-up information on any return visits (>7 days); and (8) diagnosis and procedure codes for the index encounter and all prior encounters. Considering race and ethnicity and social constructs, we will collect these data acknowledging the limitations in how these data may be reported in a retrospective study and that the means of reporting may vary by institution and over time (eg, self-reported vs determined by registration staff). These data will be reported descriptively. We will not incorporate race or ethnicity in prediction models. To minimize duplication of efforts and maximize homogeneity with data collection procedures across sites, we will promote the sharing of Structured Query Language queries using Epic and Cerner platforms across participating sites. Data will be uploaded from the EHR via a secure, HIPAA (Health Insurance Portability and Accountability Act)-compliant REDCap (Research Electronic Data Capture; Vanderbilt University) database hosted at BCM using a data upload. Data from each site will undergo quality assurance by the study team to ensure that the uploaded data from each site is free from systemic errors.

### Case Matching and Manual Data Extraction

Trained study-site PIs will abstract variables from the EHR and enter them into a secure, HIPAA-compliant REDCap database hosted at BCM. The initial upload of patient data will include data for all encounters meeting minimum inclusion criteria (age ≤90 days presenting to the ED of a participating pediatric ED with at least 1 episode of a temperature <36.5 °C during the ED encounter). Exclusions will then be applied using the methodology described earlier. Outcome data with respect to SBI and HSV will be defined by the author team using a priori defined criteria (described in the Outcome Definitions section). Cases will be identified by querying each hospital’s EHR system or microbiology laboratory in the ED during the study period to identify infants ≤90 days with SBI. Controls will include infants with a minimum rectal temperature of <36.5 °C in the ED who had a blood culture performed and with no SBI identified. We will perform 3:1 nested matching of controls to cases based on site and proximity of the date of the encounter. Among encounters where blood cultures were obtained after antibiotic administration (which may be ascertained during manual chart review), cases or controls deemed ineligible will be replaced, and case-control matching will be reperformed within that subgroup. As prior work suggests that only some infants with hypothermia have a blood culture performed, the inclusion of only those infants with a culture performed will allow for the construction of models for infants for whom a clinician may have a suspicion for SBI.

After matching cases and controls, we will provide the record numbers of these selected cases to each of the site PIs. Site PIs will manually review each encounter to ensure whether it fulfills the inclusion criteria and does not meet any exclusion criteria. If cases or controls are found to meet exclusion criteria based on manual review, new assignments for these will be provided based on the next eligible matched control based on the proximity of the encounter. Site PIs will manually abstract the following variables: (1) birth history and relevant past medical history, including gestational age, birthweight, and maternal history (including history of group B *Streptococcus* positivity, maternal fever, and use of peripartum antibiotics), admission to the neonatal intensive care unit, presence of significant medical conditions diagnosed previous to the index ED encounter, immunization status, prior treatment for hyperbilirubinemia, and sick contacts in the home; (2) presenting history (history of seizure, apnea, abnormal behavioral or lethargy, cough, congestion, vomiting, diarrhea, alteration in feeding, decreased feeding, concern for environmental exposure, presence of reported hypothermia prior to ED arrival, lowest temperature prior to ED arrival, and mode of temperature assessment); and (3) physical examination data including (a) general ill-appearance (sick, toxic, inconsolable, or meningismus), (b) poor perfusion (shock, decreased pulses, cyanotic, and pale or ashen colored), (c) mental status changes (somnolence, lethargy, and altered), (d) sunken or full fontanelle, and (e) presence of respiratory distress, apnea, cyanosis, congested, seizure, clinical bronchiolitis, jaundice, or rash on examination. Historical and physical examination findings will be categorized as present or received or absent or not received based on documentation in the EHR. Variables categorized as either undocumented or missing based on the criteria in the manual of operations.

### Compliance

All data abstractors will undergo web-based training via teleconferences and have access to a detailed manual of operation at each site. Regular updates to monitor study progress and address questions throughout the chart review process during each phase of data collection will be conducted. The REDCap database will be established according to best practices, including data validation and strategies to minimize missing data, ensuring precise data capture. Additionally, we will conduct routine monitoring and compliance reviews of the database to maintain data accuracy during the study.

### Outcome Definitions

Our primary outcome of interest is culture-confirmed SBI, defined as positive blood, urine, or CSF cultures [33-35]. Classification for pathogenic versus contaminant bacteria (from false positive cultures) will be determined based on established criteria for the classification of suspected pathogens published by the Pediatric Emergency Care Applied Research Network [36]. Indeterminate cases will be reviewed by the investigator team (which includes a specialist in pediatric infectious disease; ATC). UTI will be defined as the growth of ≥10,000 colony forming units/mL of a pathogen in urine culture plus an abnormal urinalysis (defined as the presence of leukocyte esterase, nitrites, or >5 white blood cells per high-power field on urine microscopy) [37]. We will use SBI as our outcome of interest instead of invasive bacterial infection (IBI; defined as bacteremia or bacterial meningitis and which is a preferred definition in the literature pertaining to infants with fever [26]), given the relative rarity of IBIs in previous publications and our pilot work (~2%). Cases that remain ambiguous will be reviewed by the investigator team (which includes a specialist in pediatric infectious disease; ATC), blinded to the context of relevant study predictors. We will plan an a priori sensitivity analysis limited only to infants with IBI, wherein we assess the performance of the SBI-based predictive model when using this alternative outcome. This 2-tiered approach has been previously applied to research on young infants with fever [16]. We will evaluate return visits within 7 days. For any return visits, we will review testing performed to identify potential missed cases of SBI. Our secondary outcome of interest will be HSV infection. HSV will be identified by the presence of a positive polymerase chain reaction or culture from any source [23].

### Predictors and Subgroups

We have selected candidate predictors based on a review of prior literature [4,5,9,12,15,16,38] and following discussion among members of the study team. For each predictor, we will seek to ensure that data elements can be reliably and accurately extracted from the EHR. A list of candidate predictors that we will study is provided in Table 1.

**Table 1:** Candidate predictors for the development of a multivariable model for serious bacterial infection.

Category	Candidate predictors
Past history	Gestational age, birthweight, small for gestational age status, previous use of antibiotics, previous neonatal intensive care unit hospitalization, maternal history of fever, peripartum antibiotics, group B *Streptococcus* colonization
Demographics	Age, sex, season
Presenting history	Hypothermia prior to emergency department arrival, seizure, apnea, abnormal behavior, cough, congestion, vomiting, diarrhea feeding changes, environmental exposure
Vital signs	Minimum and maximum temperature, persistent hypothermia on multiple temperature assessments, systolic blood pressure, respiratory rate, heart rate
Physical examination	Ill appearance, compromised perfusion, mental status changes, fontanelle changes, respiratory distress, apnea, seizure, congestion, jaundice, rash
Laboratory assessment	Components of complete blood count, procalcitonin, C-reactive protein, urinalysis, results of respiratory pathogen testing

### Analyses

#### Objective 1: Epidemiology of SBI and HSV

We will describe the epidemiology of SBI and HSV among infants with hypothermia prior to the selection of controls and stratify infants into groups of UTI, bacteremia without meningitis, and bacterial meningitis (with or without bacteremia). We will calculate the percentage of infants having each outcome among the overall cohort of infants with hypothermia and among the cohort of infants who had a blood culture. We will also characterize important clinical outcomes, including the proportion of infants hospitalized following their ED visit and in-hospital mortality, and describe variation in care among the included study sites.

#### Objective 2: Role of Biomarkers in the Prediction of SBI

We will calculate descriptive statistics and use the Wilcoxon rank-sum test to compare parameters among infants with and without SBI. We will evaluate the individual role of continuous laboratory predictors (including components of the CBC such as the white blood count, absolute neutrophil count, band count, and platelets) for an outcome of SBI. For these predictors, we will determine optimal cutoff values by identifying the optimal point on the receiver operator characteristic curve (as defined by the Youden index) and derive test characteristics based on the optimal cutoffs. The area under the receiver operator characteristic curve will be the empirical determinant of whether predictors are related to the likelihood of occurrence of SBI.

#### Objective 3: Derivation and Internal Validation of a Prediction Rule

We will construct a multivariable predictive model using penalized mixed effects logistic regression to identify predictors associated with SBI. We will calculate adjusted odds ratios with 95% CIs to quantify the magnitude of the association between each predictor and the outcome, considering the study site and matched group as random effects. For continuous predictors (such as age or values of blood tests), we will evaluate three approaches: (1) retaining the variable as a continuous linear predictor, (2) classifying them as categorical variables based on optimally selected points on the receiver operator characteristic curve, or (3) exploring nonlinear or nonbinary categorical effects by applying restricted cubic splines.

After the construction of the full predictive model, we will use bootstrap backward selection to develop a more parsimonious model involving fewer predictors most related to the occurrence of SBI [39]. We will assess the performance of our model using calibration metrics [40,41] and use bootstrapping for internal validation. We will report metrics of the diagnostic accuracy (sensitivity, specificity, and positive and negative likelihood ratios) of our study sample at differing decision thresholds. We will develop a risk score to best identify children who are at risk of SBI by assigning point values to included predictors to develop an easily usable point-based scoring system [38].

We will assess the performance of the predictive model by its discrimination and calibration. Discrimination will be measured by calculation of the concordance index [42] and the area under the receiver operator characteristic of the predicted probabilities of SBI generated by the models. Calibration will be evaluated by plotting the observed smoothed proportions of SBI against the predicted proportions over the observed range of predicted probabilities and by calculating the Hosmer-Lemeshow statistic [43]. We will perform bootstrap resampling to calculate indices of overfitting and overoptimism to evaluate if the model is tailored to the specific study cohort and therefore not generalizable [39]. Statistical analyses will be conducted using R (version 4.3.2; R Foundation for Statistical Computing).

### Missing Data

As with all retrospective chart reviews, there is a risk of encountering missing or inaccurately documented data. We will implement clear definitions and provide detailed instructions for data extraction and interpretation, with a particular focus on subjective data points. Additionally, we will analyze the factors associated with such missingness. These measures will help reinforce the assumption that the missing data are missing at random, thus enhancing the robustness of the study findings. We will evaluate complete case versus multiple imputation-based approaches for missing data for data elements that are missing in <20% of cases. Data missing in excess of this proportion will be excluded from multivariable modeling.

### Sample Size

We estimate identifying 41,250 infants with hypothermia in the proposed study from 25 sites over 10 years. We expect that at least 825 (2%) will have an SBI, resulting in a 95% CI width for the prevalence estimate of ±0.14%. Of these, approximately 25% (n=205) will have an IBI [5,6,14]. Greater than 80% of patients with hypothermia who have a blood culture also have a CBC performed [44]. From our sample of 41,250 infants anticipated from objective 1, we estimate that 25% (approximately n=10,310) will have a blood culture performed; and of these, 8250 will additionally have a CBC. As an exploratory component of this aim, we will evaluate inflammatory markers (specifically, CRP and procalcitonin). Prior work suggests that the use of these biomarkers is increasing over time, with 30% of patients with procalcitonin and 39.6% with CRP in 2019 [19]. Projected from our pilot data, we expect to have 650 patients with procalcitonin from our study, of whom 65 will have an SBI.

For our third objective, we anticipate having a sufficiently large sample size for the development of robust multivariable models. Under the 3:1 case-control design, the proportion of individuals with SBI in the dataset will be fixed at 0.25. Assuming 825 patients with SBI from objective 1, we estimate that the concordance index for the model will have a maximal CI half-width of 2.3%. At the expected model concordance index of roughly 0.80, the CI half-width will be 1.8%, which represents substantial precision in estimating the model’s quality of fit [45].

### Ethical Considerations

This study involves the review of medical records for patients. Institutional review board (IRB) approval as exempt from the requirements of informed consent has been obtained from the Ann & Robert H. Lurie Children’s Hospital of Chicago (IRB #2023-5779) and the 26 other participating hospitals (Table 2). Data use agreements have been established between all hospitals and BCM. All data uploaded into the REDCap repository are deidentified. No compensation is provided to participants. Adverse events, including breaches of confidentiality, will be reported to the local IRBs of Ann & Robert H. Lurie Children’s Hospital of Chicago.

**Table 2:** List of included sites.

Hospital name	City
Ann & Robert H. Lurie Children’s Hospital of Chicago	Chicago, IL
Boston Children’s Hospital	Boston, MA
Children’s Healthcare of Atlanta	Atlanta, GA
Children’s Hospital Colorado	Aurora, CO
Children’s Hospital Los Angeles	Los Angeles, CA
Children’s Hospital of Orange County	Orange, CA
Children’s Hospital of Philadelphia	Philadelphia, PA
Children’s Medical Center Dallas, UT Southwestern Medical Center	Dallas, TX
Children’s Mercy Kansas City	Kansas City, MO
Children’s Minnesota	Minneapolis and St Paul, MN
Cincinnati Children’s Hospital Medical Center	Cincinnati, OH
Hasbro Children’s Hospital	Providence, RI
Johns Hopkins Children’s Center	Baltimore, MD
Medical College of Wisconsin, Children’s Wisconsin	Milwaukee, WI
MUSC Shawn Jenkins Children’s Hospital	Charleston, SC
Nemours Children’s Health	Wilmington, DE
Norton Children’s Hospital, University of Louisville	Louisville, KY
Primary Children’s Hospital	Salt Lake City, UT
Rady Children’s Hospital-San Diego	San Diego, CA
Texas Children’s Hospital, Baylor College of Medicine	Houston, TX
UH Rainbow Babies & Children’s Hospital, Cleveland Medical Center	Cleveland, OH
UMass Memorial Children’s Medical Center, University of Massachusetts Medical School	Worcester, MA
University of Florida Health Shands Children’s Hospital	Gainesville, FL
University of Michigan Health C.S. Mott Children’s Hospital	Ann Arbor, MI
UPMC Children’s Hospital of Pittsburgh	Pittsburgh, PA
Weill Cornell Medical Center	New York, NY
Yale New Haven Children’s Hospital, Yale School of Medicine	New Haven, CT

## Results

Initial site recruitment included coinvestigators and clinicians with relevant research experience and strong engagement with the study question. As of May 1, 2025, 27 sites have been included in the study, with data uploaded to a central REDCap repository from 23 sites, with 62,771 records prior to the application of exclusion criteria. Currently, we have reviewed data from 16 sites, encompassing 45,673 infants (median age 25, IQR 6-52 days), with 9361 (20.5%) receiving blood cultures, 9120 (20%) urine cultures, and 4470 (10.4%) CSF cultures. UTIs were identified in 253 (0.6%) infants and bacteremia or meningitis in 103 (0.2%), with most IBIs being isolated bacteremia. *Escherichia coli* was the most common pathogen, and mortality among infants with SBI was 7 (2.1%), primarily among those with bacteremia or bacterial meningitis. We intend to complete data submission using automated EHR criteria by June 2025, with manual chart review completed by December 2025. We expect to submit the main study findings for publication within 9 months following the final data analysis.

## Discussion

### Overview

This study aims to identify risk factors for SBIs among young infants with hypothermia and to develop a predictive model to inform clinical decision-making. We hypothesize that a subset of infants with hypothermia, particularly those with specific clinical and laboratory features, are at increased risk for SBI and that a multivariable model can reliably stratify this risk. By leveraging a large multicenter dataset, we anticipate that our findings will support more targeted evaluation and management strategies for this vulnerable population.

### Principal Findings

Using a large, multicenter sample of infants presenting to the ED with hypothermia, this study will characterize the epidemiology of SBI and HSV and assess the diagnostic value of biomarkers in the prediction of SBI. This work will culminate in a statistical model to determine the risk of SBI among this patient population using clinical and laboratory factors and facilitate the development of a parsimonious model.

### Limitations

First, preliminary research suggests that not all infants with hypothermia receive testing for SBI in EDs and that the proportion who do receive testing varies based on the criteria used to define hypothermia [15,19,44,46]. However, this is of limited impact, as our model will allow physicians to risk-stratify children for SBI on the subset of patients for whom diagnostic testing may be considered due to a suspicion of SBI. To further investigate this potential bias, we will compare demographics among children who are tested and who are not tested for SBI. A second limitation pertains to missing data; some infants may not have documentation of relevant risk factors or data required to calculate outcomes. We will evaluate both complete case versus selective multiple imputation-based approaches for missing data, acknowledging that missingness may be related to clinical decisions, such as ordering blood tests. Third, we plan to construct a parsimonious model that is generalizable (ie, both reproducible and transportable) to best serve the needs of clinicians who manage infants with hypothermia in the ED. We will assess the internal validity of the model using bootstrap resampling techniques, from which we will derive overoptimism indexes for various measures of model fit, including the concordance index [47].

Although this will be the largest sample of infants with hypothermia in the ED, our findings may not be completely representative of the prevalence of SBI among these patients because most participating institutions are academic institutions and pediatric hospitals; therefore, the findings may not be generalizable to different care settings. However, the PEM CRC represents geographic and socioeconomically diverse areas, and we would not expect that a systematic bias would be introduced.

### Comparisons With Prior Work

The majority of prior work has been limited to single-center data and therefore is of limited generalizability [6,9,12]. Limitations of prior multicenter work include small sample size, incorporation of infants with fever within the study sample, inclusion of infants with hypothermia during their hospitalization (and not only the ED stay, where management options differ) [4,5], and lack of internal validation. We will build upon these prior studies by providing a larger, analytically robust approach to identify risk factors for SBI in this population. The proposed study will be significantly larger, focused on the initial ED presentation and decision-making, and represent a more diverse population than prior studies. It will address a continued knowledge gap in the care of young infants.

### Future Directions

The findings from this study allow for additional research that will potentially allow for the implementation of real-time risk calculation for the presence of serious infections among infants with hypothermia. Our preliminary results (which corroborate with other work [14]) suggest that SBIs occur infrequently but are associated with high mortality, heightening challenges in risk stratification in this population. Future steps may therefore be intended to safely facilitate a reduction in low-value testing. Following the completion of this study, we will seek to externally validate and implement the model via clinical decision support systems and guidelines, reducing both overtreatment in low-risk infants and health care variability, thus improving care standardization and reducing the incidence of low-value care. This will potentially lower health care costs, decrease antibiotic exposure and unnecessary hospitalization, and alleviate the emotional and financial burden on families. At the study’s conclusion, dissemination efforts will involve broad participation from key partners in pediatrics, emergency medicine, hospital medicine, primary care pediatrics, infectious disease, and dissemination or implementation science.

### Conclusions

We aim to address the critical gap in evidence-based guidance for managing hypothermia in young infants presenting to the ED. By leveraging a large, multicenter dataset, we will delineate the epidemiology of SBI and HSV in this vulnerable population, evaluate the predictive value of biomarkers, and develop a clinically useful prediction model to stratify the risk of SBI. Our findings are expected to enhance patient care by reducing unnecessary invasive procedures and antibiotic exposure. If a robust model is developed and validated externally, these results will contribute to more standardized and evidence-based care, potentially reducing health care costs and the burden on families. Despite limitations such as variability in testing and the generalizability of the findings to nonacademic settings, this research marks a significant advancement in understanding and managing hypothermia in young infants.

## Abbreviations

BCM: Baylor College of Medicine
CBC: complete blood count
CRP: C-reactive protein
CSF: cerebrospinal fluid
ED: emergency department
EHR: electronic health record
HIPAA: Health Insurance Portability and Accountability Act
HSV: herpes simplex virus
IBI: invasive bacterial infection
ICD-9: International Classification of Diseases, Ninth Revision
ICD-10: International Statistical Classification of Diseases, Tenth Revision
IRB: institutional review board
PEM CRC: Pediatric Emergency Medicine Collaborative Research Committee
PI: principal investigator
REDCap: Research Electronic Data Capture
SBI: serious bacterial infection
UTI: urinary tract infection

## Data Availability

The datasets generated or analyzed during this study are not publicly available due to institutional data use agreements and restrictions but are available from the corresponding author on reasonable request. The study’s results will be published in peer-reviewed journals and shared through conference presentations and web-based education platforms.
